# Early pregnancy biomarkers of inflammation and metabolic regulation in polycystic ovary syndrome

**DOI:** 10.1210/clinem/dgag098

**Published:** 2026-03-09

**Authors:** Simon Alesi, Taitum Mason, Stacey J Ellery, Kate Rassie, Adriana C H Neven, Yitayeh Belsti, Eveline Jona, Sharleen O’Reilly, David Simmons, Helena Teede, Aya Mousa, IMPACT DIABETES B2B&ME, David Simmons, William Hague, Helena Teede, Wah Cheung, Christopher Nolan, Michael Peek, Jeff Flack, Mark Mclean, Vincent Wong, Emily Hibbert, Alexandra Kautzky-Willer, Jürgen Harreiter, Helena Backman, Emily Gianatti, Arianne Sweeting, Viswanathan Mohan, TOBOGM Study Groups, Sharleen O’Reilly, Helena Teede, Fionnuala McAuliffe, Cristina Campoy, Christy Burden, Helle Terkildsen Maindal, Timothy Skinner, Rachel Laws, Karsten Vrangbaek

**Affiliations:** Monash Centre for Health Research and Implementation (MCHRI), Monash University, Clayton, VIC 3168, Australia; Monash Centre for Health Research and Implementation (MCHRI), Monash University, Clayton, VIC 3168, Australia; The Ritchie Centre, Hudson Institute of Medical Research and Department of Obstetrics and Gynaecology, Monash University, Clayton, Melbourne, VIC 3168, Australia; Monash Centre for Health Research and Implementation (MCHRI), Monash University, Clayton, VIC 3168, Australia; Department of Diabetes, Monash Health, Clayton, Melbourne, VIC 3168, Australia; Monash Centre for Health Research and Implementation (MCHRI), Monash University, Clayton, VIC 3168, Australia; Monash Centre for Health Research and Implementation (MCHRI), Monash University, Clayton, VIC 3168, Australia; Monash Centre for Health Research and Implementation (MCHRI), Monash University, Clayton, VIC 3168, Australia; School of Agriculture and Food Science, University College Dublin, Belfield, Dublin, Ireland; UCD Perinatal Research Centre, School of Medicine, University College Dublin, National Maternity Hospital, Dublin, Ireland; Macarthur Clinical School, Western Sydney University, Campbelltown, NSW 2560, Australia; Monash Centre for Health Research and Implementation (MCHRI), Monash University, Clayton, VIC 3168, Australia; Department of Diabetes, Monash Health, Clayton, Melbourne, VIC 3168, Australia; Monash Centre for Health Research and Implementation (MCHRI), Monash University, Clayton, VIC 3168, Australia

**Keywords:** inflammation, biomarkers, polycystic ovary syndrome (PCOS), monocyte chemoattractant protein-1 (MCP1), fatty acid binding protein (FABP4), adipokines

## Abstract

**Objective:**

To profile biomarkers of inflammation and metabolic regulation in early pregnancy among women with and without polycystic ovary syndrome (PCOS), and to assess whether these biomarkers influence the relationship between PCOS and adverse maternal or neonatal outcomes.

**Design:**

Cross-sectional study with interaction analyses using pooled baseline data from 2 international multicenter clinical trials.

**Subjects:**

One hundred and fifty-one pregnant women (at <24 weeks’ gestation) at risk of gestational diabetes mellitus, including 65 with PCOS and 86 without PCOS.

**Main outcome measures:**

Early pregnancy markers of inflammation and metabolic regulation were assessed, including C-reactive protein, tumor necrosis factor-α, interleukins, monocyte chemoattractant protein-1 (MCP1), fatty acid binding protein-4 (FABP4), growth differentiation factor-15, and adipokines (total adiponectin, leptin, resistin, chemerin, vaspin, and cathepsin-S). Biomarkers differing by PCOS status were further examined for interaction effects on maternal and neonatal outcomes.

**Results:**

Monocyte chemoattractant protein-1 concentrations were 15% higher in women with PCOS compared with those without (geometric mean ratio [GMR]: 1.15; 95% CI: 1.01, 1.31; *P* & .04), but this association was attenuated after adjustment for maternal age and body mass index (BMI). Early pregnancy FABP4 concentrations were 32% higher in women with PCOS compared with those without (GMR: 1.32; 95% CI: 1.04, 1.68; *P* & .02), and this association remained significant after adjustment for maternal age (GMR: 1.34; 95% CI: 1.05-1.71; *P* & .02), but was attenuated after adjustment for BMI. No other biomarkers differed by PCOS status, and MCP1 or FABP4 showed no interactions with maternal or neonatal outcomes.

**Conclusion:**

Monocyte chemoattractant protein-1 and FABP4 concentrations were elevated in early pregnancy among women with PCOS, largely reflecting maternal adiposity. These markers did not modify associations between PCOS and pregnancy outcomes. Larger longitudinal studies are needed to clarify underlying inflammatory and metabolic pathways.

Polycystic ovary syndrome (PCOS) is the most common endocrinological condition in women of reproductive age, affecting 10% to 13% depending on the population and diagnostic criteria used (1). Diagnosis of PCOS is based on 2023 International Guideline criteria, requiring the presence of 2 of irregular menstrual cycles; clinical or biochemical hyperandrogenism; and/or polycystic ovary morphology or elevated anti-Müllerian hormone levels (1, 2). Metabolic dysfunction is a defining feature of PCOS, characterized by obesity and insulin resistance, which together increase the risk of type 2 diabetes and cardiovascular disease in affected women (3). Psychological disorders including anxiety and depression are also common in PCOS, as are dermatological conditions such as hirsutism and acne, and reproductive problems including infertility, and pregnancy complications (1, 2).

As a chronic multisystem disorder, PCOS is associated with heightened inflammation and metabolic dysfunction, which increase the risk of cardiometabolic complications during and after pregnancy (1). These are compounded by the additional metabolic demands and proinflammatory state of pregnancy, contributing to worsened maternal and neonatal outcomes (4). Emerging evidence from preclinical and observational studies, as well as Mendelian randomization and meta-analyses from our group (5, 6) and others (7-9), has identified that specific biomarkers (such as monocyte chemoattractant protein-1 [MCP1], interleukins, and adipokines), may be involved in the pathogenesis and/or progression of PCOS. These biomarkers have also been implicated in the development of adverse pregnancy outcomes, including gestational diabetes mellitus (GDM), hypertensive disorders of pregnancy, preterm birth, and neonatal complications (10-12). While the gestational trajectories of these biomarkers remain poorly characterized, they are known to be influenced by maternal adiposity, metabolic status and age, and may contribute to immunological and metabolic adaptations in pregnancy, including changes in inflammatory signaling and insulin sensitivity (10, 13). Given their involvement in key metabolic and inflammatory pathways (14), these biomarkers may serve as early indicators of heightened pregnancy risk in women with PCOS, offering potential utility for risk stratification and early intervention.

Therefore, this study aimed to profile biomarkers of inflammation and metabolic regulation in pregnancies complicated with PCOS, and to explore whether candidate biomarkers may influence the association between PCOS and adverse maternal or neonatal outcomes.

## Materials and methods

### Source data

The current study analyzed bio-banked samples collected from participants who took part in 2 previous trials: the Treatment of Booking Gestational Diabetes Mellitus (TOBOGM) and the “Bump2Baby and Me” (B2B&Me) clinical trials (15, 16). TOBOGM was an international multicenter randomized controlled trial (RCT) and cohort study (*n* = 3672) designed to evaluate the impact of early treatment for GDM (diagnosed before 20 weeks of gestation), compared with standard care (treatment initiated at 24-28 weeks’ gestation) among women with one or more GDM risk factors (17). B2B&Me was an international multicenter single-blind RCT of 865 pregnant women investigating the impacts of a mobile health (mHealth) coaching program on weight management for those at high risk of developing GDM. All participants were aged 18 years or older, with a singleton pregnancy and at risk of GDM.

Participants from TOBOGM were recruited between 4 and 19 weeks + 6 days’ gestation, with at least one risk factor for hyperglycemia in pregnancy, including previous GDM, body mass index (BMI) > 30 kg/m^2^, first-degree relative with diabetes, age ≥40 years, previous macrosomic infant, PCOS, or non-European ancestry. Women who were unable to understand English or had any active medical disorders were excluded.

For B2B&Me, women were recruited at <24 weeks’ gestation and screened for GDM risk using the validated Monash GDM screening tool (18). This tool incorporates maternal age, BMI, ethnicity, family history of diabetes, previous GDM, and recent poor obstetric outcome; and those with a score of ≥3 were eligible for inclusion (18). Exclusion criteria included established diabetes (type 1 or type 2), current multiple pregnancy, malignant cancer, inability to verbally communicate, severe mental illness, substance abuse, or myocardial infarction within the 3 months before recruitment.

### Ethics

The TOBOGM study was pre-registered on the Australian New Zealand Clinical Trials Registry (ACTRN12616000924459) and received ethical approval from the Southwestern Sydney Local Health District Human Research and Ethics Office, Australia (Ref: 15/LPOOL/551) (19). Local approval from the Monash Health Research Ethics Committee was obtained for recruitment and data collection at the Monash site and for this substudy (Ref: RES-17-0000-406X). The B2B&Me study was pre-registered on the Australian New Zealand Clinical Trials (ACTRN12620001240932) and received ethical approval for all study sites, as detailed elsewhere (15). Local approval from the Monash Health Research Ethics Committee was obtained for recruitment and data collection at the Monash site and for this substudy (Ref: RES-20-0000-892A). All participants provided informed written consent prior to participation.

### Substudy design: combined cohort

This substudy was designed *a priori* and included in the original ethics submissions for the main trials as described above. For TOBOGM, 130 participants from the Monash cohort were included. Participant selection comprised all participants reporting a PCOS diagnosis (*n* = 63) and a random sample without PCOS (*n* = 67). For B2B&Me, 22 participants from the Monash cohort were selected, capturing those with PCOS (*n* = 2) and without (*n* = 19), with one participant excluded because their PCOS status was unknown (*n* = 1). Therefore, a total combined sample of 151 women sampled at baseline (*n* = 65 with PCOS and *n* = 86 without PCOS) were included in this substudy. The final number of samples analyzed was determined by available funding and laboratory capacity, with the aim of prioritizing all available PCOS cases for inclusion and assessing a broad panel of biomarkers within this exploratory framework. To retain PCOS cases and explore the influence of covariates, a matched design was not used; instead, potential confounding was addressed analytically using multivariable modeling.

### Demographic, clinical, and anthropometric measures

For participants in both the TOBOGM and B2B&Me trials, demographic, clinical, and anthropometric measures for the current analysis were self-reported on questionnaires or measured by study investigators at the first (baseline) clinical visit (<24 weeks’ gestation, conducted between January 2018 and December 2021). Questionnaires included self-reported demographic characteristics (eg, age, ethnicity), PCOS status (self-reported as having been previously diagnosed and/or having PCOS symptoms), and medical history (eg, previous GDM, family history of diabetes, previous obstetric complications). Maternal weight and height were measured at the baseline visit using calibrated scales; these were used to calculate BMI as kg/m^2^. Blood pressure was also measured at this visit using a calibrated blood pressure monitor.

### Biochemical measures and laboratory analysis

All blood samples were collected at the baseline visit following an overnight fast. A range of biomarkers were assessed at this early pregnancy timepoint using Luminex multi-analyte kits (R&D Systems, USA). These included markers of inflammation: C-reactive protein (CRP), tumor necrosis factor-alpha (TNF-α), interleukins (IL)-1β/6/8 and MCP1; and markers of metabolic regulation: fatty acid binding protein-4 (FABP4), growth differentiation factor-15 (GDF15), chemerin, vaspin (serpin A12), leptin, total adiponectin (hereafter referred to as “adiponectin’), resistin, and cathepsin-S. All laboratory analyses were conducted in duplicate, and the mean value was used to calculate final concentrations. Where duplicate measurements showed notable variation, assay performance was evaluated against plate calibrators and assay coefficient of variation thresholds to confirm reliability. Standard calibrator samples were included on every plate to control for batch effects and enabled calculation of intra- and inter-assay coefficients of variation (CV), which ranged from 4.6% to 17.6% and 2.2% to 16.4%, respectively.

### Maternal and neonatal outcomes

Maternal and neonatal outcomes were obtained from hospital records and included pregnancy-induced hypertension (composite of preeclampsia, eclampsia, or gestational hypertension); preterm birth (delivery at <37 completed weeks’ gestation); antepartum hemorrhage; and perineal injury (20). Although GDM was assessed in these cohorts (using the World Health Organization 2013 criteria [21]), it was not included as an outcome in this substudy, given the variations in terms of early diagnosis in the TOBOGM study and treatment initiation for some women. Neonatal outcomes included birth trauma (20), congenital anomaly, large- or small-for-gestational age (LGA/SGA; defined as birthweight > 90th or <10th percentile based on ethnic group- and sex-adjusted customized percentiles, respectively), jaundice/phototherapy, hypoglycemia (heel-prick glucose ≤ 40 mg/dL [≤ 2.2 mmol/L] at 1-2 hours after birth), sepsis, respiratory distress (requiring ≥ 4 hours of supplemental oxygen, continuous positive airway pressure, intermittent positive-pressure ventilation, or combinations thereof, during the first 24 hours post-birth), shoulder dystocia (obstetrical maneuvres performed during vaginal birth to deliver the fetus after delivery of the head and failed gentle traction), and admission to a neonatal intensive care unit (NICU).

### Statistical analysis

All statistical analyses were conducted in STATA SE18 (College Station, TX: StataCorp LLC.). Normality of continuous variables was evaluated through histograms. Demographic, anthropometric, and clinical characteristics were summarized as mean ± SD or median with interquartile ranges (IQRs) for normally and non-normally distributed continuous variables, respectively. Categorical variables were reported as counts (*n*) and percentages. Differences in these variables by PCOS status were assessed using independent samples t-tests for normally distributed continuous data, Mann–Whitney U tests for non-normally distributed continuous data, and χ^2^ tests for categorical variables.

Biomarkers were naturally logarithmically transformed to normalize skewed distributions and to mitigate the effects of outliers (22). For the purposes of interpretation, we report geometric means with 95% confidence intervals, as biomarker concentrations typically follow a log-normal distribution, with skewness driven by extreme high values if this skewness is not due to bias (23). Univariable and multivariable linear regression analyses were performed where each log biomarker concentration is the outcome and PCOS status is the binary predictor. Covariates included maternal age and BMI, with additional sensitivity analyses adjusting for study source and for gestational age at sampling. These covariates were selected as potential confounders, given their known associations with both PCOS status and inflammatory/metabolic regulation, as well as their influence on pregnancy outcomes. Other demographic variables, including ethnicity, were not included as covariates due to limited sample size and to reduce the risk of collinearity and overfitting. Regression coefficients for PCOS were exponentiated to derive geometric mean ratios (GMRs) with 95% confidence intervals, estimating the multiplicative difference in biomarkers between women with and without PCOS. A GMR >1 indicates higher biomarker concentrations in women with PCOS.

Early pregnancy biomarkers showing a statistically significant association in linear regression were then carried forward into interaction analyses to assess relationships with maternal and neonatal outcomes. Mediation analysis was conducted within a counterfactual framework; however, due to rare outcomes and data separation, reliable estimates could not be obtained. Consequently, Firth's penalized logistic regression was used to assess associations and potential interactions between candidate early pregnancy biomarkers, PCOS status, and adverse maternal and neonatal outcomes. Firth's penalized logistic regression is equipped to reduce small-sample bias and obtain finite estimates in the presence of sparse cells/possible separation (24). Results are presented as odds ratios with profile-likelihood 95% confidence intervals. As a sensitivity analysis, standard maximum-likelihood logistic regression yielded similar conclusions. Statistical significance was defined as a 2-tailed *P* value <.05 for all analyses. Given the exploratory nature of this substudy, a formal *a priori* power calculation was not performed. Accordingly, analyses were not adjusted for multiple testing, and all results are considered hypothesis-generating.

## Results

### Sample characteristics

Participant characteristics for this substudy cohort (*n* = 151) are presented in Table 1. Participants were enrolled and sampled at a median of 14.5 (IQR: 12.0-16.5) weeks’ gestation, with all but 3 (98%) recruited prior to 20 weeks’ gestation. The mean age was 33.2 ± 4.4 years, and the cohort was ethnically diverse, including individuals identifying as South Asian (40.4%), Caucasian (30.5%), Southeast Asian (11.3%), East Asian (6.6%), Middle Eastern (2.7%), Aboriginal (0.7%), Pacific Islander (0.7%), and other backgrounds (7.3%).

**Table 1:** Baseline data at early pregnancy for overall cohort and by PCOS status

Characteristic	All (*n* = 151)	PCOS (*n* = 65)	No PCOS (*n* = 86)	*P*
**Age (years)**	33.2 ± 4.4	33.5 ± 3.6	33.7 ± 4.9	.10
**Ethnicity, *n* (%)**				
Caucasian	46 (30.5)	26 (40.0)	20 (23.3)	.**044**
Middle Eastern	4 (2.7)	3 (4.6)	1 (1.2)	
South Asian	61 (40.4)	26 (40.0)	35 (40.7)	
South East Asian	17 (11.3)	4 (6.2)	13 (15.1)	
East Asian	10 (6.6)	1 (1.5)	9 (10.5)	
Aboriginal	1 (0.7)	1 (1.5)	0 (0.0)	
Pacific Islands	1 (0.7)	0 (0.0)	1 (1.2)	
Other	11 (7.3)	4 (6.2)	7 (8.1)	
**Gestation (weeks)**	14.5 (12.0, 16.5)	13.0 (12.0, 16.0)	16.0 (12.0,17.0)	.**0043**
**Anthropometry**				
Weight (kg)	75.5 ± 24.0	80.6 ± 27.2	71.7 ± 20.6	.**024**
BMI (kg/m^2^)	28.5 ± 8.1	30.3 ± 9.2	27.1 ± 6.9	.**018**
**Blood pressure**				
Systolic Blood Pressure (mmHg)	111.1 ± 10.7	111.2 ± 11.1	111.2 ± 10.6	.98
Diastolic Blood Pressure (mmHg)	67.5 ± 9.1	66.9 ± 10.1	68.1 ± 8.4	.48
**Family history of T2DM, *n* (%)**				
Yes	59 (60.7)	33 (38.8)	26 (40.0)	.88
No	91 (39.3)	52 (61.2)	39 (60.0)	
**Parity, *n* (%)**				
Null	65 (42.8)	30 (46.2)	34 (39.5)	.45
1	60 (39.5)	22 (33.9)	38 (44.2)	
2-3	26 (17.1)	13 (20.0)	13 (15.1)	
>3	1 (0.7)	0 (0.0)	1 (1.2)	
**Hormones**				
Total testosterone (ng/mL)	1.3 ± 0.7	1.5 ± 0.7	1.2 ± 0.6	.**045**
5-α dihydrotestosterone (pg/mL)	136.7 (79.1, 189.3)	167.8 (105.7, 192.9)	121.2 (66.1, 170.5)	.**0031**
Androstenedione (ng/mL)	2.5 (1.7, 3.5)	2.9 (1.9, 3.8)	2.3 (1.7, 3.4)	.06
Dehydroepiandrosterone (ng/mL)	4.4 (3.2, 5.9)	4.5 (3.2, 5.7)	4.4 (3.0, 5.9)	.78
**Inflammation markers** *^a^*				
CRP (mg/L)	10.0 (8.7, 11.5)	11.5 (8.7, 11.5)	9.2 (7.5, 11.3)	.12
MCP1 (pg/mL)	121.7 (114.1, 129.8)	131.9 (119.9, 145.1)	114.9 (105.1, 125.5)	.**038**
TNF-α (pg/mL)	2.1 (1.9, 2.3)	2.2 (1.8, 2.5)	2.0 (1.8, 2.3)	.55
IL1β (pg/mL)	5.6 (5.2, 6.1)	5.9 (5.3, 6.7)	5.4 (4.8, 6.1)	.28
IL6 (pg/mL)	1.7 (1.6, 1.8)	1.8 (1.6, 2.0)	1.6 (1.4, 1.8)	.19
IL8 (pg/mL)	3.7 (3.2, 4.2)	4.1 (3.4, 5.0)	3.4 (2.8, 4.2)	.20
**Metabolic regulation markers** *^a^*				
Chemerin (pg/mL)	2910.9 (2669.4, 3174.3)	3066.7 (2709.2, 3471.4)	2804.9 (2477.6, 3175.6)	.33
GDF15 (pg/mL)	3221.3 (2921.4, 3551.9)	3260.2 (2823.4, 3764.6)	3185.3 (2769.3, 3663.7)	.82
FABP4 (pg/mL)	2581.8 (2291.4, 2909.0)	3039.4 (2505.3, 3687.5)	2298.7 (1973.0, 2678.1)	.**024**
Leptin (pg/mL)	9872.5 (8918.2, 10928.9)	10506.0 (8977.0, 12295.5)	9493.4 (8270.0, 10897.8)	.34
Resistin (pg/mL)	6482.2 (6005.5, 6996.7)	6615.0 (5925.9, 7384.3)	6346.5 (5690.6, 7077.9)	.60
Vaspin (serpin A12) (pg/mL)	291.9 (233.5, 364.9)	325.4 (231.8, 456.8)	271.1 (199.1, 369.1)	.43
Adiponectin (pg/mL)	24958.1 (23559.8, 26439.4)	25185.7 (22993.3, 27587.0)	24770.3 (22915.1, 26775.7)	.78
Cathepsin-S (pg/mL)	1554.6 (1440.2, 1678.1)	1470.2 (1297.3, 1666.1)	1621.7 (1470.2, 1788.7)	.21

Data are expressed as mean ± standard deviation for normally distributed data or median (interquartile range) for non-normal distributions; categorical data are expressed as frequencies (*n*, %).

^*a*^Biomarker data are presented as geometric means, with 95% confidence intervals. Differences by PCOS status were analyzed using Mann–Whitney U or independent samples t-tests depending on normality for continuous variables and a χ^2^ test for categorical variables. For inflammatory biomarker analysis, the raw values were naturally logarithmically transformed prior to analysis, and analyzed using independent samples t-tests. Numbers may vary due to missing data. Two-tailed *P* < .05 was considered statistically significant, comparing women with PCOS vs non-PCOS, denoted in bold.

Abbreviations: BMI, body mass index; CRP, C-reactive protein; FABP4, fatty acid binding protein 4; GDF15, growth differentiation factor-15; IL, interleukin; MCP1, monocyte chemoattractant protein-1; PCOS, polycystic ovary syndrome; TNF-α, tumor necrosis factor-α.

At baseline, women with PCOS had higher body weight, BMI, total testosterone, and 5-alpha dihydrotestosterone (DHT) levels; different ethnic composition; and an earlier gestation at their first visit (median 13 weeks) compared with those without PCOS (median 16 weeks). There were no differences in other demographic or clinical parameters between PCOS and non-PCOS groups (Table 1).

### Differences in early pregnancy biomarkers by PCOS status

Early pregnancy biomarker concentrations in PCOS and non-PCOS groups are presented in Tables 1 and 2, with pregnancy outcomes shown in Tables 3-5. In pairwise comparisons, MCP1 and FABP4 concentrations were significantly higher in women with PCOS compared with controls (*P* = .04 and *P* = .02, respectively; Table 1). Univariable linear regression showed similar results, whereby MCP1 geometric mean concentrations were 15% higher in women with PCOS compared with non-PCOS (GMR 1.15, 95% CI 1.01, 1.31; *P* = .04) and FABP4 geometric mean concentrations were 32% higher in PCOS compared with non-PCOS (GMR 1.32, 95% CI 1.04, 1.68; *P* = .02; Table 2).

**Table 2:** Univariable and multivariable differences in early pregnancy biomarkers by PCOS status

Log Biomarker	Univariable				Model 1*^a^*				Model 2*^b^*			
	*n*	GMR	95% CI	*P*	*n*	GMR	95% CI	*P*	*n*	GMR	95% CI	*P*
**Inflammation markers**												
CRP	150	1.25	0.95, 1.63	.11	150	1.31	1.00, 1.71	.05	150	1.13	0.89, 1.45	.32
TNF-α	150	1.06	0.87, 1.30	.56	150	1.08	0.89, 1.32	.44	150	1.07	0.89, 1.27	.49
MCP1	151	1.15	1.01, 1.31	.**04**	151	1.14	1.00, 1.30	.05	151	1.10	0.97, 1.25	.15
IL1β	143	1.10	0.93, 1.30	.26	143	1.12	0.94, 1.32	.20	143	1.10	0.92, 1.31	.30
IL6	130	1.12	0.95, 1.31	.17	130	1.14	0.97, 1.33	.12	130	1.05	0.91, 1.21	.52
IL8	132	1.21	0.92, 1.58	.17	132	1.21	0.93, 1.59	.16	132	1.14	0.83, 1.56	.43
**Metabolic regulation markers**												
FABP4	150	1.32	1.04, 1.68	.**02**	150	1.34	1.05, 1.71	.**02**	150	1.17	0.94, 1.44	.16
GDF15	137	1.02	0.84, 1.25	.82	137	1.03	0.85, 1.26	.75	137	1.05	0.85, 1.29	.62
Leptin	150	1.11	0.90, 1.36	.33	150	1.10	0.89, 1.35	.40	150	0.97	0.79, 1.18	.73
Adiponectin	150	1.02	0.90, 1.14	.78	150	1.02	0.91, 1.15	.69	150	1.06	0.94, 1.20	.31
Chemerin	149	1.09	0.92, 1.30	.31	149	1.06	0.89, 1.26	.53	149	1.03	0.85, 1.23	.78
Resistin	148	1.04	0.90, 1.21	.59	148	1.05	0.90, 1.22	.54	148	1.03	0.88, 1.20	.75
Vaspin (Serpin A12)	150	1.20	0.76, 1.88	.43	150	1.27	0.80, 2.00	.31	150	1.34	0.85, 2.11	.20
Cathepsin-S	151	0.91	0.78, 1.06	.22	151	0.88	0.76, 1.03	.11	151	0.88	0.75, 1.02	.10

All continuous biomarker data were naturally logarithmically transformed to address non-normality and reduce the influence of skewed distributions. Univariable and multivariable linear regression models were used to examine differences in log biomarker concentrations by PCOS status, with the biomarker as the outcome and PCOS as the binary predictor. Exponentiated regression coefficients are presented as geometric mean ratios (GMRs) with 95% confidence intervals, which reflect the multiplicative difference in biomarker levels between women with and without PCOS. GMR > 1 indicates higher levels in PCOS vs non-PCOS. Differences in sample sizes reflect missing covariate data and/or missing multiplex data due to the set dilution(s). Two-tailed *P* < .05 was considered statistically significant, denoted in bold.

^*a*^The model was adjusted for maternal age.

^*b*^The model was adjusted for maternal age and body mass index.

Abbreviations: CRP, C-reactive protein; FABP4, fatty acid binding protein 4; GDF15, growth differentiation factor-15; GMR, geometric mean ratio; IL, interleukin; MCP1, monocyte chemoattractant protein-1; TNF-α, tumor necrosis factor-α.

**Table 3:** Maternal and neonatal outcomes for the overall cohort and by PCOS status

Pregnancy outcome	All (*n* = 151)	PCOS (*n* = 65)	No PCOS (*n* = 86)	*P*
NICU admission	27/140 (19.3%)	14/56 (25.0%)	13/84 (15.5%)	.16
Neonate jaundice	26/141 (18.4%)	10/56 (17.9%)	16/85 (18.8%)	.89
Perineal injury	82/139 (59.0%)	49/83 (59.0%)	33/56 (58.9%)	.99
Neonate respiratory distress	31/140 (22.1%)	15/56 (26.8%)	16/84 (19.1%)	.28
SGA	7/122 (5.7%)	2/39 (5.1%)	5/83 (6.0%)	.84
LGA	20/123 (16.3%)	4/39 (10.3%)	16/84 (19.1%)	.22
Birth injury	12/141 (8.5%)	5/56 (8.9%)	7/85 (8.2%)	.89
Congenital anomaly	5/142 (3.5%)	2/57 (3.5%)	3/75 (3.5%)	.99
Neonate hypoglycemia	30/132 (22.7%)	12/53 (22.6%)	18/79 (22.8%)	.99
Neonate sepsis	6/138 (4.4%)	4/56 (7.1%)	2/82 (2.4%)	.18
Preterm birth	11/143 (7.7%)	6/59 (10.2%)	5/84 (6.0%)	.35
Shoulder dystocia	3/141 (2.1%)	1/56 (1.8%)	2/85 (2.4%)	.82
Pregnancy-induced hypertension	4/141 (2.8%)	1/56 (1.8%)	3/85 (3.5%)	.54
Antepartum hemorrhage	7/142 (4.9%)	3/57 (5.3%)	4/85 (4.7%)	.88

Data are expressed as frequencies (*n*, %). Differences by PCOS status were analyzed using χ^2^ tests. Numbers may vary due to missing values. Two-tailed *P* < .05 was considered statistically significant, comparing women with PCOS vs non-PCOS.

Abbreviations: LGA, large for gestational age; NICU, neonatal intensive care unit; PCOS, polycystic ovary syndrome; SGA, small for gestational age.

**Table 4:** Interaction between early pregnancy MCP1, PCOS status, and maternal and neonatal outcomes

Outcome	Effect	Univariable			Model 1*			Model 2**		
		OR	95% CI	*P*	aOR	95% CI	*P*	aOR	95% CI	*P*
NICU admission	No PCOS	1.16	0.66, 2.01	.61	1.17	0.67, 2.04	.59	1.12	0.63, 1.99	.70
	PCOS	1.78	0.89, 3.54	.10	1.77	0.89, 3.51	.10	1.47	0.73, 2.97	.28
	Interaction	1.54	0.64, 3.72	.34	1.51	0.62, 3.67	.36	1.31	0.54, 3.42	.55
Neonatal Jaundice	No PCOS	1.15	0.69, 1.92	.59	1.18	0.70, 1.99	.54	1.16	0.69, 1.97	.57
	PCOS	1.10	0.54, 2.24	.80	1.09	0.54, 2.22	.80	1.03	0.50, 2.14	.93
	Interaction	0.95	0.40, 2.30	.92	0.93	0.38, 2.25	.87	0.89	0.37, 2.15	.79
Perineal Injury	No PCOS	1.14	0.74, 1.73	.56	1.07	0.68, 1.67	.78	1.11	0.70, 1.75	.65
	PCOS	1.07	0.61, 1.86	.82	1.06	0.60, 1.87	.85	1.23	0.68, 2.22	.50
	Interaction	0.94	0.47, 1.89	.86	0.99	0.48, 2.04	.98	1.10	0.53, 2.31	.79
Neonatal Respiratory Distress	No PCOS	1.27	0.76, 2.13	.37	1.24	0.74, 2.08	.41	1.25	0.75, 2.08	.40
	PCOS	1.53	0.80, 2.93	.20	1.53	0.80, 2.93	.20	1.52	0.76, 3.03	.24
	Interaction	1.21	0.53, 2.77	.66	1.23	0.54, 2.83	.62	1.22	0.52, 2.85	.65
SGA	No PCOS	1.11	0.48, 2.60	.80	1.14	0.48, 2.74	.78	1.12	0.46, 2.73	.81
	PCOS	8.19	0.56, 118.94	.12	10.21	0.49, 213.00	.13	7.75	0.37, 160.48	.19
	Interaction	7.35	0.44, 121.75	.16	8.94	0.39, 205.03	.17	6.95	0.32, 152.20	.22
Birth injury	No PCOS	2.31	1.13, 4.72	.**02**	2.27	1.10, 4.66	.**03**	2.82	1.06, 7.48	.**04**
	PCOS	1.09	0.42, 2.80	.87	1.08	0.42, 2.78	.87	2.55	0.47, 13.99	.28
	Interaction	0.47	0.14, 1.54	.21	0.48	0.15, 1.57	.22	0.91	0.14, 5.87	.92
Congenital anomaly	No PCOS	1.88	0.74, 4.76	.19	1.81	0.71, 4.62	.22	1.80	0.74, 4.40	.20
	PCOS	0.55	0.14, 2.18	.39	0.55	0.14, 2.16	.40	0.58	0.15, 2.31	.44
	Interaction	0.29	0.06, 1.55	.15	0.31	0.06, 1.59	.16	0.32	0.06, 1.65	.18
LGA	No PCOS	1.15	0.69, 1.92	.59	1.28	0.73, 2.23	.39	1.11	0.60, 2.05	.74
	PCOS	1.38	0.45, 4.24	.57	1.45	0.45, 4.70	.54	1.16	0.35, 3.87	.80
	Interaction	1.20	0.35, 4.12	.77	1.14	0.31, 4.17	.85	1.05	0.27, 4.02	.95
Neonatal hypoglycemia	No PCOS	1.27	0.78, 2.06	.34	1.27	0.78, 2.08	.34	1.26	0.77, 2.06	.35
	PCOS	0.94	0.49, 1.82	.87	0.94	0.49, 1.82	.86	0.89	0.45, 1.77	.74
	Interaction	0.75	0.33, 1.69	.48	0.74	0.33, 1.68	.48	0.71	0.31, 1.62	.41
Neonatal sepsis	No PCOS	1.83	0.61, 5.47	.28	1.68	0.57, 4.93	.35	1.67	0.59, 4.69	.33
	PCOS	0.80	0.29, 2.24	.68	0.81	0.29, 2.29	.69	0.84	0.28, 2.54	.76
	Interaction	0.44	0.10, 1.98	.28	0.48	0.11, 2.16	.34	0.50	0.11, 2.29	.38
Preterm birth	No PCOS	2.15	0.99, 4.67	.05	2.03	0.94, 4.41	.07	2.02	0.93, 4.37	.07
	PCOS	1.23	0.50, 2.99	.65	1.22	0.51, 2.96	.65	1.13	0.45, 2.81	.79
	Interaction	0.57	0.17, 1.86	.35	0.60	0.19, 1.95	.40	0.56	0.17, 1.85	.34
Shoulder dystocia	No PCOS	1.45	0.44, 4.84	.54	1.31	0.41, 4.16	.65	1.25	0.37, 4.20	.72
	PCOS	2.97	0.35, 25.33	.32	2.71	0.42, 17.59	.30	1.96	0.30, 12.91	.49
	Interaction	2.04	0.17, 23.89	.57	2.07	0.23, 18.76	.52	1.56	0.17, 14.34	.69
PIH	No PCOS	1.34	0.48, 3.76	.57	1.30	0.47, 3.60	.61	1.18	0.39, 3.56	.76
	PCOS	0.37	0.06, 2.16	.27	0.38	0.06, 2.18	.28	0.32	0.05, 1.93	.21
	Interaction	0.27	0.04, 2.12	.21	0.29	0.04, 2.16	.23	0.27	0.03, 2.13	.21
Antepartum hemorrhage	No PCOS	2.31	1.00, 5.33	.05	2.35	0.99, 5.62	.05	2.33	0.99, 5.47	.05
	PCOS	1.45	0.43, 4.95	.55	1.43	0.42, 4.93	.57	1.44	0.39, 5.30	.59
	Interaction	0.63	0.14, 2.77	.54	0.61	0.13, 2.76	.52	0.62	0.13, 2.95	.55

Firth's penalized logistic regression with a product interaction term was used to examine the interaction between early pregnancy monocyte chemoattractant protein-1 (MCP1), polycystic ovary syndrome (PCOS) status, and adverse maternal and neonatal outcomes. All continuous biomarker values were natural log-transformed and standardized (z-score) to address non-normality. Estimates are presented as odds ratios (ORs) per 1-SD increase in log-MCP1 with 95% confidence intervals (CIs). **“**No PCOS” and **“**PCOS” rows report group-specific MCP1 effects derived from linear combinations of main and interaction terms. Differences in sample sizes across models reflect missing biomarker or covariate data. Two-tailed *P*-values <.05 were considered statistically significant, denoted in bold. *Model 1 was adjusted for maternal age. **Model 2 was adjusted for maternal age and body mass index.

Abbreviations: a/OR, adjusted/odds ratio; CI, confidence interval; LGA, large for gestational age; MCP1, monocyte chemoattractant protein-1/C-C motif chemokine ligand 2; PCOS, polycystic ovary syndrome; PIH, pregnancy-induced hypertension; SGA, small for gestational age.

**Table 5:** Interaction between early pregnancy FABP4, PCOS status, and maternal and neonatal outcomes

Outcome	Effect	Univariable			Model 1*			Model 2**		
		OR	95% CI	*P*	aOR	95% CI	*P*	aOR	95% CI	*P*
NICU admission	No PCOS	0.98	0.54, 1.79	.95	0.98	0.54, 1.78	.94	0.83	0.44, 1.55	.55
	PCOS	1.41	0.77, 2.57	.27	1.40	0.76, 2.55	.28	1.06	0.55, 2.05	.87
	Interaction	1.43	0.61, 3.37	.41	1.43	0.61, 3.34	.41	1.28	0.55, 2.97	.57
Neonatal jaundice	No PCOS	1.44	0.82, 2.52	.21	1.43	0.81, 2.50	.22	1.54	0.85, 2.79	.15
	PCOS	1.95	0.94, 4.05	.08	1.93	0.93, 4.02	.08	2.18	0.96, 4.98	.06
	Interaction	1.35	0.54, 3.41	.52	1.36	0.54, 3.41	.52	1.41	0.55, 3.65	.47
Perineal injury	No PCOS	0.82	0.52, 1.29	.39	0.84	0.52, 1.34	.46	0.93	0.57, 1.52	.77
	PCOS	0.93	0.56, 1.55	.78	0.95	0.56, 1.63	.86	1.14	0.63, 2.06	.66
	Interaction	1.14	0.57, 2.25	.71	1.14	0.56, 2.33	.72	1.23	0.60, 2.54	.57
Neonatal respiratory distress	No PCOS	0.77	0.43, 1.37	.37	0.77	0.43, 1.38	.39	0.74	0.41, 1.36	.34
	PCOS	0.97	0.55, 1.71	.92	0.98	0.56, 1.72	.94	0.89	0.48, 1.66	.71
	Interaction	1.27	0.56, 2.85	.57	1.26	0.56, 2.84	.57	1.20	0.53, 2.68	.67
SGA	No PCOS	1.37	0.47, 5.98	.43	1.36	0.56, 3.28	.50	1.28	0.50, 3.26	.61
	PCOS	1.37	0.56, 3.33	.49	1.69	0.47, 6.08	.42	1.46	0.38, 5.69	.58
	Interaction	1.23	0.26, 5.79	.80	1.25	0.26, 5.88	.78	1.14	0.25, 5.21	.86
Birth injury	No PCOS	1.08	0.49, 2.35	.85	1.09	0.50, 2.36	.84	1.46	0.64, 3.30	.37
	PCOS	0.48	0.19, 1.20	.12	0.49	0.20, 1.21	.12	0.81	0.30, 2.24	.69
	Interaction	0.45	0.13, 1.48	.19	0.45	0.14, 1.48	.19	0.56	0.15, 2.02	.38
Congenital anomaly	No PCOS	1.84	0.60, 5.69	.29	1.83	0.60, 5.58	.29	2.25	0.68, 7.46	.18
	PCOS	0.73	0.20, 2.74	.64	0.74	0.19, 2.81	.66	0.93	0.21, 4.04	.92
	Interaction	0.40	0.07, 2.25	.30	0.40	0.07, 2.29	.31	0.41	0.07, 2.54	.34
LGA	No PCOS	1.54	0.87, 2.71	.14	1.52	0.85, 2.72	.16	1.16	0.61, 2.21	.65
	PCOS	2.76	0.91, 8.34	.07	2.82	0.88, 9.10	.08	1.81	0.51, 6.42	.36
	Interaction	1.80	0.52, 6.22	.36	1.86	0.50, 6.88	.35	1.56	0.39, 6.20	.53
Neonatal hypoglycemia	No PCOS	0.98	0.58, 1.66	.94	0.98	0.58, 1.66	.95	0.99	0.57, 1.72	.98
	PCOS	1.28	0.69, 2.39	.44	1.28	0.69, 2.38	.44	1.27	0.64, 2.52	.49
	Interaction	1.30	0.58, 2.95	.52	1.30	0.58, 2.93	.52	1.28	0.56, 2.89	.56
Neonatal sepsis	No PCOS	1.19	0.30, 4.66	.80	1.20	0.31, 4.70	.80	1.33	0.31, 5.63	.70
	PCOS	0.72	0.28, 1.88	.51	0.74	0.28, 1.96	.55	0.82	0.27, 2.44	.72
	Interaction	0.61	0.11, 3.22	.56	0.62	0.12, 3.32	.58	0.61	0.11, 3.37	.57
Preterm birth	No PCOS	1.36	0.56, 3.32	.50	1.38	0.56, 3.35	.48	1.43	0.56, 3.65	.45
	PCOS	1.57	0.67, 3.68	.30	1.58	0.68, 3.65	.29	1.60	0.61, 4.19	.34
	Interaction	1.15	0.34, 3.95	.82	1.15	0.34, 3.89	.83	1.12	0.33, 3.84	.86
Shoulder dystocia	No PCOS	2.72	0.66, 11.15	.17	2.99	0.62, 14.38	.17	2.51	0.47, 13.53	.28
	PCOS	1.29	0.21, 7.90	.78	1.31	0.29, 5.91	.72	1.06	0.20, 5.51	.95
	Interaction	0.48	0.05, 4.73	.53	0.44	0.05, 3.93	.46	0.42	0.05, 3.38	.42
PIH	No PCOS	1.12	0.36, 3.45	.85	1.13	0.37, 3.42	.83	0.89	0.27, 2.91	.85
	PCOS	2.57	0.41, 16.25	.32	2.61	0.42, 16.18	.30	1.60	0.25, 10.18	.62
	Interaction	2.30	0.26, 20.03	.45	2.32	0.27, 19.67	.44	1.80	0.24, 13.78	.57
Antepartum hemorrhage	No PCOS	0.85	0.31, 2.36	.75	0.85	0.31, 2.36	.76	0.83	0.29, 2.38	.72
	PCOS	0.82	0.27, 2.47	.72	0.82	0.27, 2.47	.72	0.75	0.23, 2.39	.62
	Interaction	0.97	0.21, 4.37	.97	0.96	0.21, 4.29	.95	0.90	0.21, 3.91	.89

Firth's penalized logistic regression with a product interaction term was used to examine the interaction between early pregnancy fatty acid binding protein 4 (FABP4), polycystic ovary syndrome (PCOS) status, and adverse maternal and neonatal outcomes. All continuous biomarker values were natural log-transformed and standardized (z-score) to address non-normality. Estimates are presented as odds ratios (ORs) per 1-SD increase in log-FABP4 with 95% confidence intervals (CIs). “No PCOS” and “PCOS” rows report group-specific MCP1 effects derived from linear combinations of main and interaction terms. Differences in sample sizes across models reflect missing biomarker or covariate data. Two-tailed *P*-values <.05 were considered statistically significant. *Model 1 was adjusted for maternal age. **Model 2 was adjusted for maternal age and body mass index.

Abbreviations: a/OR, adjusted/odds ratio; CI, confidence interval; FABP4, fatty acid binding protein-4; LGA, large for gestational age; PCOS, polycystic ovary syndrome; PIH, pregnancy-induced hypertension; SGA, small for gestational age.

In multivariable regression, differences in MCP1 by PCOS status were attenuated by adjustment for age (Model 1) and BMI (Model 2; Table 2). Geometric mean concentrations of FABP4 remained 34% higher in PCOS compared with non-PCOS in the age-adjusted model (Model 1; GMR 1.34; 95% CI 1.05, 1.71; *P* = .02); however, this was attenuated upon adjustment for BMI (Model 2; Table 2). For both MCP1 and FABP4, sensitivity analyses with additional adjustment for study source or gestational age at sampling did not alter the results (Table S1 (25)).

No significant differences were observed in other inflammation markers including CRP, TNF-α, and IL concentrations, or in other metabolic regulation markers including GDF-15, leptin, adiponectin, resistin, chemerin, vaspin (serpin A12) or cathepsin-S, between PCOS and non-PCOS groups (Tables 1 and 2). Sensitivity analyses with additional adjustment for study source or for gestational age at sampling did not alter the results (Table S1 (25)).

### Interaction analysis for early pregnancy biomarkers, PCOS, and maternal and neonatal outcomes

Early pregnancy biomarkers that differed significantly between groups were carried forward to penalized Firth's logistic regression analysis to examine their interactions with PCOS status and adverse maternal and neonatal outcomes. Pregnancy outcomes across the cohort and by PCOS status are shown in Table 3, with no significant differences between groups. As shown in Table 4, there were no statistically significant interaction effects observed between MCP1 and PCOS for any pregnancy outcome across univariable, age-adjusted (Model 1), or fully adjusted (Model 2) analyses. Among women without PCOS, higher MCP1 levels were associated with increased odds of birth injury (OR 2.31; 95% CI 1.13, 4.72; *P* = .02), which remained significant after adjustment for maternal age (aOR 2.27; 95% CI 1.10, 4.66; *P* = .03) and BMI (aOR 2.82; 95% CI 1.06, 7.48; *P* = .04). There were no statistically significant interaction effects observed between FABP4 and PCOS for any pregnancy outcome across univariable, age-adjusted (Model 1), or fully adjusted (Model 2) analyses (Table 5). These results were materially unchanged in sensitivity analyses with additional adjustment for study source or gestational age at sampling (Tables S2 and S3 (25)).

## Discussion

This study provides a comprehensive evaluation of inflammatory and metabolic biomarkers in early pregnancy among women with and without PCOS. This is, to our knowledge, the first study to investigate novel metabolic biomarkers including GDF15, FABP4, vaspin (serpin A12), and cathepsin-S in this context. Using harmonized baseline data from 2 international multicenter clinical trials, we demonstrated that women with PCOS had elevated early pregnancy concentrations of MCP1 and FABP4 compared with their non-PCOS counterparts; however, these associations were attenuated after adjusting for maternal BMI. Additionally, interaction analysis showed that these biomarkers did not influence the relationship between PCOS and adverse maternal or neonatal outcomes.

Among the biomarkers examined, MCP1 and FABP4 were significantly higher in pregnant women with PCOS compared with the non-PCOS group on univariable analyses. Monocyte chemoattractant protein-1 functions as a key chemokine that regulates monocyte recruitment and macrophage activation. It has been implicated in the pathogenesis of several chronic conditions, including rheumatoid arthritis and cardiovascular disease (7, 12, 26, 27), as well as in adverse pregnancy outcomes such as spontaneous abortion (28), preeclampsia (29, 30), and preterm labor (31). Our findings align with accumulating evidence from preclinical models, observational cohorts, genetic approaches such as Mendelian randomization, and recent meta-analyses, indicating that MCP1 contributes to the inflammatory-metabolic dysregulation characteristic of PCOS. Similarly, FABP4 is an adipokine predominantly expressed in adipocytes and macrophages, where it regulates intracellular lipid trafficking, modulates insulin signaling, and promotes inflammation (32). It has been linked with obesity, insulin resistance, type 2 diabetes, cardiovascular disease, as well as PCOS in nonpregnant cohorts (33, 34); however, no previous studies have assessed FABP4 in PCOS pregnancies. Importantly, for both MCP1 and FABP4, the differences observed were attenuated after adjustment for BMI, suggesting that elevated levels might primarily reflect maternal adiposity and related metabolic factors rather than PCOS itself. This aligns with a 2021 meta-analysis of 11 studies among nonpregnant women (*n* = 897; 529 PCOS and 368 non-PCOS), which reported higher MCP1 concentrations in PCOS overall, but no differences within subgroups stratified into women with obesity and PCOS vs controls with obesity only, or lean women with PCOS vs lean controls (7). Similarly, FABP4 elevations in PCOS appear to reflect underlying metabolic dysfunction in PCOS, particularly adiposity and insulin resistance, though evidence for an independent association with PCOS remains inconclusive (33).

Despite higher concentrations in PCOS, neither MCP1 nor FABP4 mediated the relationship between PCOS and adverse maternal or neonatal outcomes. It should be noted, however, that circulating levels of MCP1 and FABP4 may not accurately reflect their local activity at the maternal–fetal interface, where both chemokine and adipokine signaling within the trophoblast, decidua, and placenta are likely to be more biologically relevant (12). Overall, our results suggest that systemic markers, such as MCP1 and FABP4, reflect the broader influence of adiposity on inflammatory and metabolic pathways but are unlikely to be pathogenic drivers of adverse outcomes in PCOS pregnancies.

Our findings broadly align with prior evidence that women with PCOS exhibit a chronic low-grade inflammatory state (35); yet, we did not observe widespread elevations across multiple inflammatory markers. In contrast, the large multicenter study by Stokkeland et al (35), which similarly examined early gestation, reported higher CRP, IL-6, IL-18, TNF-α, and MCP1 concentrations in women with PCOS. Another study showed that women with PCOS (*n* = 150) had higher CRP and white blood cell (WBC) counts across all gestational timepoints, compared with age- and BMI-matched healthy pregnant controls (*n* = 150), which were associated with greater risk of adverse obstetric outcomes in the PCOS group (36). The absence of significant group differences for most inflammatory markers in our study likely reflects population characteristics, as our cohort comprised women already at high metabolic risk, reducing contrast between groups and potentially requiring a larger sample to detect subtle differences. Heterogeneity in PCOS, ethnicity, and the multifactorial nature of inflammatory signaling may also contribute to discrepancies between studies (37), as may methodological variation in assay type and sensitivity (37).

Similarly, no significant differences by PCOS status were observed for other metabolic markers and adipokines measured, including GDF15, adiponectin, leptin, resistin, chemerin, vaspin (serpin A12), and cathepsin-S. This should be interpreted cautiously, as the modest sample sizes and the subtle, multifactorial nature of inflammatory signaling in early gestation may limit the ability to detect small but biologically relevant effects. While some of these markers, such as leptin and adiponectin, have been examined previously in PCOS pregnancies, others, including vaspin (serpin A12), GDF15, and cathepsin-S, have not been examined in this context. Growth differentiation factor-15 has been associated with hyperemesis gravidarum, placental size, and early pregnancy loss (38, 39), while vaspin and cathepsins have been implicated in ovarian and placental dysfunction (40-42), and in processes involved in labor and delivery (43). However, there is no evidence regarding the roles of these markers in PCOS pregnancies. Similarly, elevated concentrations of chemerin, a proinflammatory adipokine, were associated with early miscarriage in a small Chinese cohort of PCOS pregnancies (*n* = 58) (44). It may also be a potential therapeutic target in PCOS (45), although evidence in PCOS pregnancies remains sparse. Longitudinal studies with larger, well-powered sample sizes and repeated measures of these biomarkers across gestation are warranted to clarify their potential relevance in PCOS pregnancies.

### Strengths and limitations

This study provides a comprehensive evaluation of markers of inflammation and metabolic regulation in early pregnancy among women with and without PCOS including, for the first time, exploration of novel metabolic biomarkers such as GDF15, FABP4, vaspin (serpin A12), and cathepsin-S in this context. We include a well-characterized and ethnically diverse cohort, enhancing the generalizability of our findings. The inclusion of early pregnancy sampling provides valuable insights into inflammatory pathways at a critical stage of gestation, before many obstetric complications develop. Biomarker measurements were performed using standardized Luminex multi-analyte assays with stringent quality controls, and statistical analyses employed log transformations to account for skewed distributions, ensuring robust and reliable estimates. In addition, the application of penalized logistic regression incorporating interactions enabled exploration of potential mechanistic pathways linking PCOS, biomarkers, and maternal-neonatal outcomes, extending beyond simple correlation analyses.

Several limitations are acknowledged, most notably the cross-sectional design, which precludes causal inference. The cohort was selected for being at high risk of GDM, with a substantial proportion affected, adding heterogeneity and complicating interpretation of PCOS-specific effects. It also comprised women who successfully conceived, potentially underrepresenting those with severe PCOS-related infertility and limiting generalizability across the full clinical spectrum of PCOS. Data on preconception PCOS treatments were not systematically collected across cohorts and therefore could not be incorporated into our analyses. Gestational diabetes mellitus was not assessed as an outcome, as differences in timing of diagnosis and treatment (early vs standard care) across the pooled trials precluded reliable comparisons and could generate potentially misleading estimates. Moreover, while serum biomarker concentrations provide useful information on systemic inflammatory and metabolic status, these may not fully capture local activity at the maternal–fetal interface, and the selected biomarker panel may not reflect the full spectrum of relevant mediators. Further, the multi-analyte kits used for biomarker quantification have not been specifically validated in pregnant populations with PCOS, raising the possibility of measurement variability. Compared with single-analyte ELISAs, multiplex platforms offer efficiency but can have lower sensitivity and specificity for certain markers and potential cross-reactivity between analytes could reduce accuracy. These methodological factors should be considered when interpreting absolute biomarker concentrations. Sample sizes for some outcomes were limited, particularly in interaction analyses, where sparse data and rare events may have led to unstable estimates or data separation. Lack of significant interaction effects should therefore be interpreted cautiously, as this may reflect limited power rather than true absence of effect modification. Although Firth's penalized logistic regression was used to reduce small-sample bias, residual imprecision remains possible. Finally, our use of pooled data enhanced statistical power, but may have introduced unmeasured variability due to differences in recruitment criteria and study protocols. However, this is unlikely to have materially affected our findings, as sensitivity analysis with adjustment for study source did not alter the results. Replication in larger, adequately powered studies is warranted to confirm these findings and further clarify the complex interplay between PCOS, adiposity, and inflammatory-metabolic pathways in pregnancy.

## Conclusions

In summary, MCP1 and FABP4 were elevated in early pregnancy among women with PCOS, but this association was largely explained by maternal adiposity, and there was no evidence that these markers influenced adverse maternal or neonatal outcomes. Although MCP1 and FABP4 may indicate metabolic dysregulation and inflammation in PCOS pregnancies, future prospective studies with large sample sizes and repeated measures are needed to clarify if these are PCOS-specific alterations or obesity-related metabolic effects, and to determine their utility as predictors of obstetric risk.

## Data Availability

The data included in this study are derived from participants enrolled in the TOBOGM and B2B&Me clinical trials and include clinical and biomarker data collected under ethics approvals. De-identified data may be made available from the corresponding author upon reasonable request, subject to approval by the relevant trial governance committees and institutional ethics requirements, and completion of a data access agreement.

## Clinical Trial Information

TOBOGM: ACTRN12616000924459; B2B&Me: ACTRN12620001240932.
